# Politics and the Doctor

**Published:** 1912-03-09

**Authors:** 


### POLITICS AND THE DOCTOR.

The chronicles of Louis the Eleventh of France relate that that astute Sovereign did not despise the counsels of his barber, his jester, his soothsayer, and his physician in deciding great matters of policy. They even relate that measures of paramount importance to the welfare of the nation which he ruled, on the whole so wisely, were initiated by the medical gentleman who had the honour—closely allied to a misfortune—to cure the King's migraine. History records other cases where medical men have made use of their interest, their knowledge, and their art to alter the character of "that eternal Turkish jig," as Dekker phrased it, which we call politics. And it says much for the sagacity of the profession that in general these efforts have been in favour of measures of policy which posterity has judged very kindly. Ambroise Paré could not succeed in preventing the night massacre, but there is evidence to show that his influence materially softened the rigorous measures adopted against his reputed co-religionists. Desault could not prevent the Terror, but there is equal evidence to prove that he saved, where his silent acquiescence might have endangered his own life. It is only in modern times that the doctor does not deem it his duty to be an active politician when he has the opportunity of influencing the character of the dance. We suppose that medical science, as we like to call it, is too sacrosanct to permit its votaries to indulge in the vain pleasures of legislation. For that reason our Parliament has the smallest percentage of medical men among its members that any legislative assembly shows. It would be difficult, indeed, for any member of the medical profession to give off-hand the name of any colleague who has ever occupied the proud position of a Minister of the Crown in this country. Abroad medical men have been Prime Ministers, Presidents, and leaders of great social movements. With us every medical man follows too keenly the advice which the *Quarterly* gave to young Keats—get back to the gallipots and stick to them.

Perhaps the introduction of the National Insurance Act—that gigantic Krakatoa of discord—may move the profession to take a more active part in the duties of citizenship. It has at least roused the profession to become interested in an experiment of social legislation which concerns the whole community. It has united the profession, or the greater part of it, in defence of what it deems first principles. Whether such union at such a time and for such an object, with the Act itself imperfectly understood, is entirely justified or not, the fact remains that the doctor has learned that he has an influence in politics, and that he can, on occasion, make that influence felt. Because this special example of social legislation has touched his pocket, threatened his practice, and plucked from him his patients, he has bethought him of that influence. The thought is tardy, but it is none the less welcome, for it is inconceivable that, once having felt the importance of the power of collective opposition which he wields, he will be content to remain a passive spectator in the future when the Turkish jig is performed. There are many matters which do not materially concern him, many figures of the dance which have as little interest for him as they have for the rest of the community. These he may safely leave to the attention of the professional politician. But there are other vital considerations which he cannot and dare not neglect. His recent experiment in politics will be productive of far more benefit to the nation, if it is extended to other fields of social legislation. The programme embraced by the word social legislation is a long one, and the doctor should have the first say in the manner of its shaping.

